# Characteristics of telemedicine workflows in nursing homes during the COVID-19 pandemic

**DOI:** 10.1186/s12913-023-09249-2

**Published:** 2023-03-29

**Authors:** James H Ford, Sally A Jolles, Dee Heller, Christopher Crnich

**Affiliations:** 1 Social & Administrative Sciences Division, University of Wisconsin School of Pharmacy, 777 Highland Ave., Madison, WI 53705 USA; 2 University of Wisconsin School of Medicine & Public Health, Madison, WI USA; 3 William S. Middleton VA Hospital, Madison, WI USA

**Keywords:** Telemedicine, Workflow, Nursing Homes, SEIPS

## Abstract

**Background:**

The use of telemedicine increased dramatically in nursing homes (NHs) during the COVID-19 pandemic. However, little is known about the actual process of conducting a telemedicine encounter in NHs. The objective of this study was to identify and document the work processes associated with different types of telemedicine encounters conducted in NHs during the COVID-19 pandemic.

**Methods:**

A mixed methods convergent study was utilized. The study was conducted in a convenience sample of two NHs that had newly adopted telemedicine during the COVID-19 pandemic. Participants included NH staff and providers involved in telemedicine encounters conducted in the study NHs. The study involved semi-structured interviews and direct observation of telemedicine encounters and post-encounter interviews with staff and providers involved in telemedicine encounters observed by research staff. The semi-structured interviews were structured using the Systems Engineering Initiative for Patient Safety (SEIPS) model to collect information about telemedicine workflows. A structured checklist was utilized to document steps performed during direct observations of telemedicine encounters. Information from interviews and observations informed the creation of a process map of the NH telemedicine encounter.

**Results:**

A total of 17 individuals participated in semi-structured interviews. Fifteen unique telemedicine encounters were observed. A total of 18 post-encounter interviews with 7 unique providers (15 interviews in total) and three NH staff were performed. A 9-step process map of the telemedicine encounter, along with two microprocess maps related to encounter preparation and activities within the telemedicine encounter, were created. Six main processes were identified: encounter planning, family or healthcare authority notification, pre-encounter preparation, pre-encounter huddle, conducting the encounter, and post-encounter follow-up.

**Conclusion:**

The COVID-19 pandemic changed the delivery of care in NHs and increased reliance on telemedicine services in these facilities. Workflow mapping using the SEIPS model revealed that the NH telemedicine encounter is a complex multi-step process and identified weaknesses related to scheduling, electronic health record interoperability, pre-encounter planning, and post-encounter information exchange, which represent opportunities to improve and enhance the telemedicine encounter process in NHs. Given public acceptance of telemedicine as a care delivery model, expanding the use of telemedicine beyond the COVID-19 pandemic, especially for certain NH telemedicine encounters, could improve quality of care.

**Supplementary Information:**

The online version contains supplementary material available at 10.1186/s12913-023-09249-2.

## Background

The COVID-19 pandemic had an extensive impact on how healthcare was delivered in almost all care settings. This was especially true in nursing homes (NHs) where residents were more susceptible to impacts of the COVID-19 pandemic [1–3]. Despite evidence demonstrating its benefits [4–6], telemedicine utilization in NHs [7, 8] prior to COVID-19 had remained disappointingly low. Research suggests that prior to the COVID-19 pandemic that the adoption of telemedicine in Nursing Homes ranged from 13 to 39% [9, 10]. The Center for Medicare and Medicaid Services (CMS) implemented sweeping policy and regulatory changes during COVID-19 in an effort to promote greater telemedicine use and curtail face-to-face clinical encounters in NH settings [11, 12]. Unsurprisingly, telemedicine use in United States NHs expanded during the COVID-19 pandemic [7, 13]. Specifically, recent estimates suggest that 84% of Nursing Homes have fully or partially implemented telemedicine post pandemic [7]. Much of the prior work on telemedicine in NHs has focused on its impact on reducing hospitalization [9, 10], its potential for expanding resident access to sub-specialty care services [4, 14], and regulatory/policy barriers to its greater use in NHs [10, 15, 16]. While there has been some work examining pre-conditions for successful deployment of a NH telemedicine program from the perspective of the clinical staff [17] and telemedicine work system enhancements [18], robust descriptions of how telemedicine encounters are conducted in NHs are lacking. Consequently, the objective of the current study is to characterize the telemedicine work system in a convenience sample of NHs that had newly adopted telemedicine using a system engineering framework.

## Methods

### Study design and setting

We conducted a mixed methods convergent study [19] of telemedicine use in NHs located in South Central Wisconsin. We approached two NHs that had newly adopted telemedicine during the COVID-19 pandemic and invited them to participate in the study. One nursing home is a for-profit organization that is part of a group of NHs and is licensed for 104 beds. The second NH is a stand-alone, not-for profit organization that is licensed for 85 beds. Both facilities are located in a Midwestern state in the United States.

The study involved semi-structured interviews with NH staff and providers involved in telemedicine visits as well as direct patient and provider observations of telemedicine encounters. Interviews with staff and providers focused on their perceptions of how telemedicine visits were conducted under routine circumstances while observations of telemedicine encounters allowed the research team to observe how the steps involved in telemedicine visits were actually conducted. In this study, the term telefacilitator refers to the NH staff member who was present and facilitated the telemedicine encounter. While the telefacilitator in study NHs often had a clinical background this was not always the case.

### Interview partcipants and data collection

The facility Director of Nursing, Medical Director, resident care managers, social workers, and providers involved in facility telemedicine encounters were purposively recruited to participate in semi-structured interviews. The purpose of the interviews was to gather general information about the structure and process of telemedicine encounters in study NHs and the challenges facilities encountered with implementing and using telemedicine during the COVID-19 pandemic.

Three interview guides, based on participate role, were developed for advance practice providers and physicians (Appendix A), NH staff (Appendix B), and members of the NH administration, including the Director of Nursing, Administrator, and Medical Director (Appendix C). They were developed using the Systems Engineering Initiative for Patient Safety (SEIPS) model [20]. The SEIPS model is an extension of the classic Donabedien structure-process-outcome model [21] that explicitly examines the characteristics and interactions between the organization, person, tools, tasks, and environment.

Members of the research team conducted observations of telemedicine encounters using a structured observation checklist (Appendix D) based on the SEIPS model. Research staff used the observation checklist to document the key people, tools, tasks, and organizational as well as environmental factors involved during different phases of the telemedicine process. Each observational checklist was completed by at least two study staff when observing a telemedicine encounter.

Verbal informed consent was obtained from NH staff and providers before interviews were conducted. Interviews were audio recorded and transcribed for analysis purposes. For telemedicine encounter observations, verbal informed consent was obtained from NH providers, NH staff, as well as residents and resident family members before research staff began documentation of the encounter. No telemedicine encounters were audio or video recorded.

### Data analysis

**Fig. 1:**
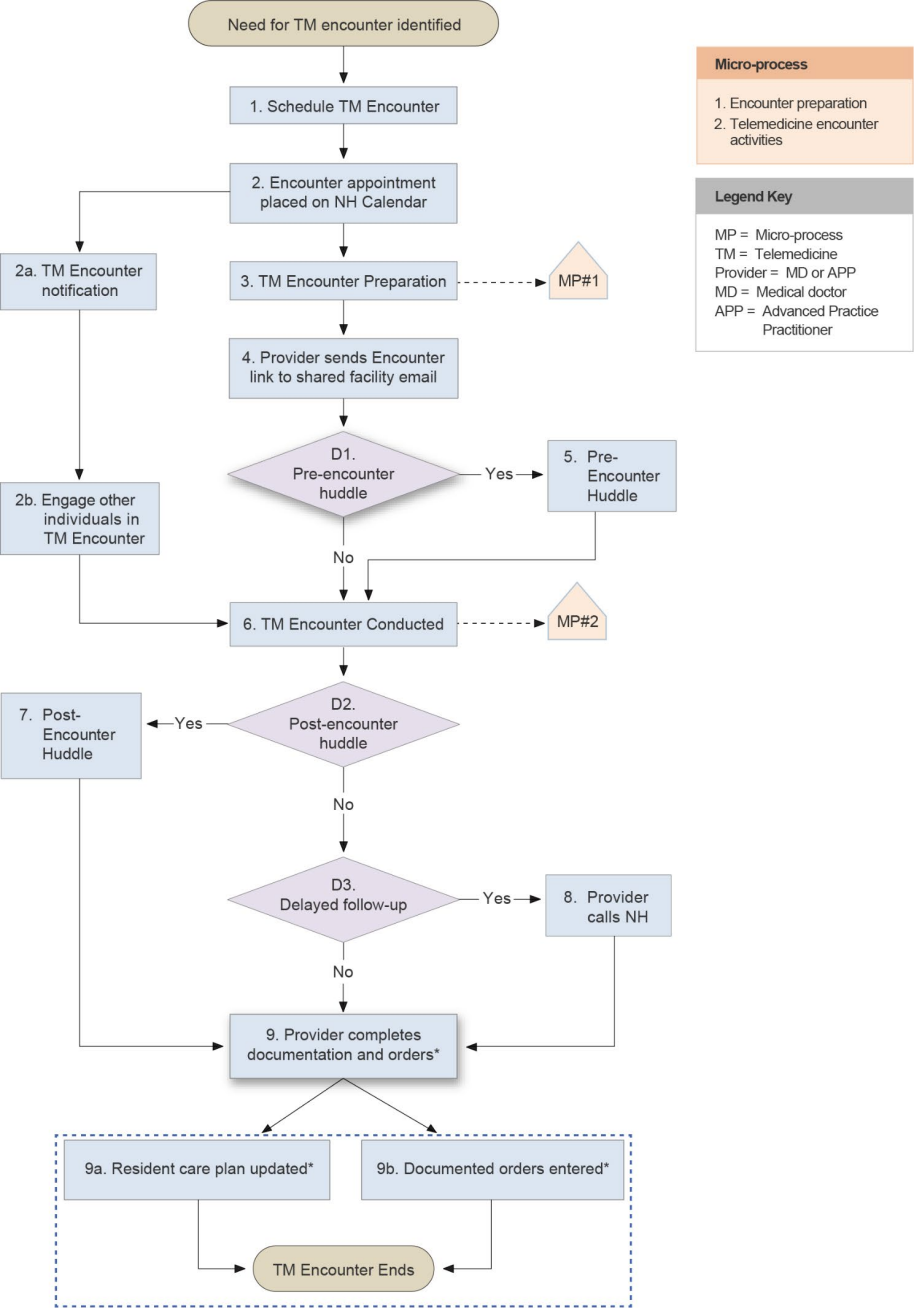
Overview of the general telemedicine encounter process in nursing homes

**Fig. 2:**
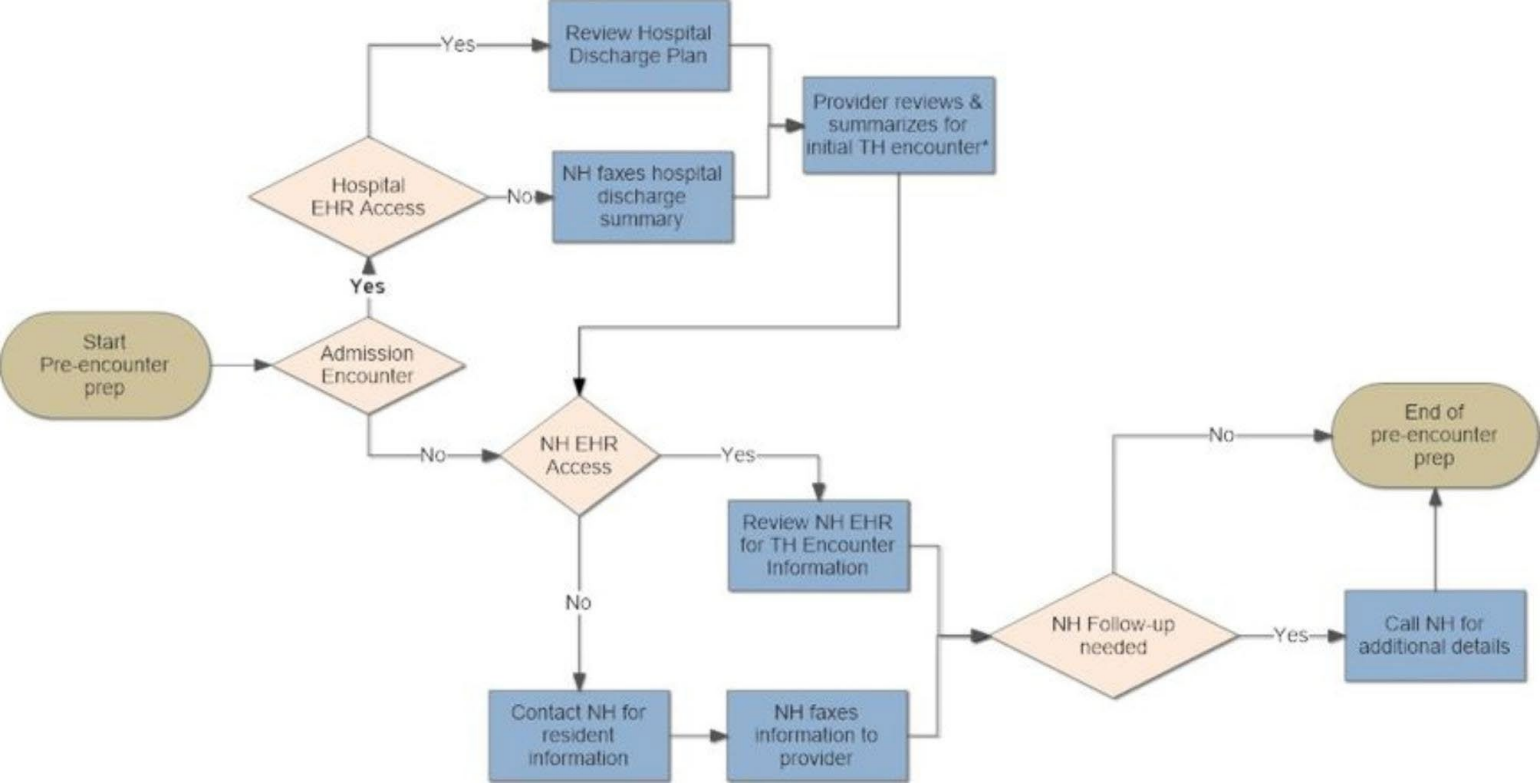
Process associated with the pre-encounter preparation

**Fig. 3:**
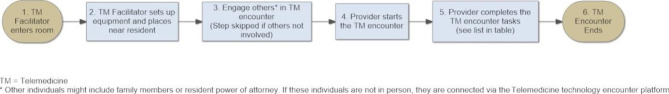
Telemedicine encounter

Participant interviews were audio recorded and transcribed. The research team utilized a directed content analysis approach [22] to independently code interview transcripts in teams of two (CC, DH, JF, SJ) based on the Systems Engineering Initiative for Patient Safety (SEIPS) model. This model was employed to identify and characterize the barriers and challenges with conducting telemedicine encounters in participating NHs. The study Principal Investigator (PI) (CC) resolved any coding discordance. Study staff observing telemedicine encounters participated in meetings to discuss discrepancies identified on checklists completed for each observation in an attempt to achieve consensus; the study PI (CC) resolved discordant results when consensus between involved study staff was not achieved. Information from consensus checklists was entered into a database and aggregated across NH sites. The core research team conducted an independent analysis of both the interview data and the observational data. Following four data integration meetings, the research team integrated and merged [22] the qualitative and quantitative results to generate a flow map of the telemedicine work-system and its microprocesses within study NHs (Figs. 1, 2 and 3).

## Results

A total of 17 individuals participated in semi-structured interviews. Ten interviews were conducted at NH A. Interviewees included providers (n = 4), NH staff (n = 3), NH administration (n = 2), and one person from the business office. A total of 7 interviews were conducted at NH B, including providers (n = 3), NH administration (n = 3), and one NH staff member. We observed 15 unique telemedicine encounters (Table 1) and identified pre- and post-telemedicine encounter activities (Table 2). Information in these tables includes data from the observational checklist. The majority (n = 7) of the encounters were for a new admission to the NH. The resident care manager served as the telefacilitator in 53.3% (n = 8) of the encounters and a a member of the medical records staff served as telefacilitator in the remaining observed encounters (n = 7, 46.7%). A total of 18 post-encounter interviews were conducted with providers (n = 15) and NH staff (n = 3).

**Table 1:** Demographics of observed telemedicine encounters (n = 15)

	Number of Observations (%)
Type of Telemedicine Encounter	
Admission Discharge Compliance Not sure	7 (46.7%) 2 (13.3%) 2 (13.3%) 4 (26.6%)
Training of staff member facilitating telemedicine encounter	
Medical Records Staff Nursing Home Resident Care Manager	7 (46.7%) 8 (53.3%)
Telemedicine encounter activities performed by facilitator ^1^	
Managed the environment for optimal quality of the visit and comfort/focus of the resident Shared history not offered by the resident with the provider Facilitated bi-directional communication between provider and resident Assisted with or performed aspects of the physical exam Coordinating follow-up communication with other NH staff and/or taking resident care orders Ensuring the PPE protocols were observed Activities related to managing, securing, setting up, or troubleshooting the telemedicine equipment Other activities (e.g., training staff)	9 (64.3%) 8 (53.3%) 7 (50.0%) 7 (50.0%) 6 (42.9%) 5 (35.7%) 4 (28.6%) 3 (21.4%)
Encounter Engagement	
Resident actively involved asking and responding to questions Resident only responding to questions Family or POA Engagement	10 (66.7%) 4 (26.7%) 3 (20.0%)

1. Persons facilitating telemedicine encounters often performed more than one activity during the observed visit

**Table 2:** Pre and post telemedicine encounter activities

	Number of Observations (%)
Pre-Huddle Occurred	7 (46.7%)
Pre-Huddle Activities (n = 7)	
Review of resident medications Care coordination/planning Discussion of Active Medical Problem or Resident Status Review of Labs New orders conveyed by the provider Review of resident vital signs Psycho-social assessment Other (e.g., family involvement, hospice visit, discharge projection)	5 (71.4%) 5 (71.4%) 3 (42.8%) 3 (42.8%) 3 (42.8%) 2 (28.6%) 2 (28.6%) 3 (42.8%)
Post Encounter Follow-up	
Delayed follow-up (provider called back) Post-Huddle Occurred (Immediately After)	10 (66.7%) 2 (13.3%)
Delayed Follow-up – Planned Provider Activities (n = 10)	
Discuss other care coordination Submit orders for resident Gather additional information	9 (90.0%) 6 (60.0%) 4 (40.0%)

Note: Data was collected on 15 telemedicine encounters

Analysis of interview transcripts and observation checklists revealed multiple steps in the NH telemedicine process. The initial step began with identifying the need for a telemedicine encounter and concluded with the completion of the telemedicine encounter (Fig. 1). The multiple steps identified are described in greater detail below.

### Scheduling the encounter

The scheduling of a telemedicine appointment (Fig. 1 - Step 1) in study NHs was a complex non-sequential process that was dependent on completion of tasks by providers or their clinic staff as well as NH staff. The process of who initiates scheduling depends on the NH telehealth appointment type. For example, the providers’ office initates encounter appointments for planned/routine visits; however, NH staff initiate appointments when an urgent or acute resident change in condition occured (Step 1). Once the need for a telemedicine encounter was initiated, finalizing selection of an appointment day/time often involved iterative back and forth between the NH and providers’ clinic (Table 3). Once the appointment date was finalized, NH staff placed the date of telemedicine encounter on the internal master calendar (Fig. 1 – Step 2).

**Table 3:** Provider vs. nursing home (NH) scheduling tasks for a telemedicine encounter

Provider Scheduling Tasks	Nursing Home (NH) Staff Scheduling Tasks
Provider or clinic staff determines availability for telemedicine visit	NH staff checks master calendar to determine staff availability to facilitate the visit
Provider availability communicated with NH*	NH confirms that equipment is available for the visit
Provider and NH coordinate time for telemedicine visit*	NH checks patient schedule to determine availability
	NH works with provider to determine time for the telemedicine visit*
	NH places telemedicine visit on master calendar

* Interdependent process which often involves multiple calls between the NH and the provider clinic

### Notification of family or healthcare power of attorney

Resident family members or the healthcare power of attorney (HCPOA) were involved in approximately 20% of the encounters observed during the study. Involvement of family members and/or HCPOA allowed these individuals to share knowledge about how their loved one was doing and receive information and participate in the resident care planning. However, involving these individuals in telemedicine encounters added considerable complexity to the encounter scheduling process. In order to involve family members or the HCPOA in a telemedicine encounter, NH staff had to notify these individuals in advance of the scheduled appointment (Step 2a), conduct a telephone reminder on the day of the encounter, forward these participants the electronic link for accessing the virtual encounter (Step 2b), and ensure these participants were connected to the virtual portal before the provider commenced with the encounter (Step 3 in Microprocess #2 of Fig. 3).

### Pre-encounter preparation

Provider preparation for the telemedicine encounter (Fig. 1 - Step 3) was dependent on the type of encounter (admission versus non-admission encounter) and ability of the provider to remotely access resident health information (Fig. 2). The hospital discharge summary was a critical piece of information needed to successfully complete NH admission encounters. Preparation for admission telemedicine encounters was more efficient when providers had remote access to the referring hospital electronic health record (EHR). Considerable coordination between provider clinic staff and NH staff was required to receive a faxed copy of the discharge summary when remote access of the hospital EHR was not possible. Providers with remote access to the NH EHR were able to independently prepare for telemedicine encounters without involving NH staff in most cases. However, preparation for telemedicine encounters when the provider lacked remote NH EHR access usually involved telephone conversations with NH staff and/or fax transmittal of pertinent records to their clinic prior to the conduct of a telemedicine encounter. Similar to scheduling a telemedicine appointment, this type of information exchange often involved multiple back and forth contacts depending on availability of the provider, clinic staff, and pertinent NH staff. Even when contact was successful, providers reported the quality of information exchanged was lower than that obtained by direct review of the NH health record. An additional complexity of faxing information to providers, who often conducted their encounters from home during the COVID-19 pandemic, centered on ensuring confidentiality of resident private health information, appropriate destruction of this information after use and ensuring the appropriate information was transmitted by the NH.

### Pre-encounter huddle

A huddle between the provider and a member of the NH staff was often (~ 50% of observed encounters) performed prior to the telemedicine encounter (Fig. 1 - Step 5) during which information about resident health status and pertinent elements of the resident care plan were discussed (Table 2). These encounters were typically conducted using the same equipment (tablet or laptop computer) employed during the conduct of the telemedicine encounter and was more likely to occur when the NH staff member facilitating the encounter was a clinician.

### Conducting telemedicine encounter

Upon receipt of the telemedicine encounter link from the provider, NH staff obtained the equipment (e.g., tablet or laptop computer) needed in order to conduct the telemedicine encounter and if necessary, ensured that family members or the HCPOA wishing to attend the appointment had connected prior to the start of the actual encounter (Fig. 1 - Step 2b). After conducting a pre-encounter huddle, if performed, the telemedicine encounter started when the telefacilitator enters the resident room to set up the equipment (Step 2 in Microprocess #2 of Fig. 3). Once the link was established, the provider had a conversation with the resident and/or their family or HCPOA about their symptoms, any change in condition, performed pertinent aspects of that exam and discussed treatment recommendations and, if appropriate, alterations to the existing care plan. These tasks varied by the residents condition and acuity. During the actual encounter, it was observed that the telefacilitator would perform different activities to support the provider (Table 1). The most common clinical activities involved sharing of history not offered by the resident with the provider (n = 8), facilitating bi-directional communication between the resident and provider (n = 7), and assisting with or performing aspects of the physicial exam (n = 7). In 66.7% of the encounters, the resident was actively engaged in asking and responding to questions.

### Post-encounter huddle and follow-up

A huddle between the provider and the telefacilitator to exchange information, provide care and treatment orders, and coordinate additional care occurred after 13% of the observed encounters (Fig. 1 - Step 7). Factors limiting the conduct of post-encounter huddles included back-to-back scheduling of other telemedicine encounters, conflicts with other nursing work-related activities, and limited availability of the bedside nurse who was often the only person with the information required by the provider conducting a particular telemedicine encounter. In this latter situation, the provider would conduct a delayed telephone follow-up (Fig. 1 – Step 8) with the bedside nurse in order to obtain the needed information. While post-encounter huddles were used to convey resident care orders, delayed telephone follow-up (67% of observations) occurred when the provider needed to review additional information (e.g., test results), or consult with other members of the NH staff (e.g., physical therapist) or family members/HCPOA. The breakdown of activities performed during delayed telephone follow-up are described in Table 2.

## Discussion

The COVID-19 pandemic greatly expanded the use of telemedicine encounters in NHs [7, 13]. The current study utilized the SEIPS model to describe the steps of the telemedicine encounter revealing that it is a complex multistep process. The SEIPS model has been employed to identify human factors involved in the implementation of telemedicine encounters in NHs during the COVID-19 pandemic [17] and identify work system enhancements in NHs that had newly adopted telemedicine [18]. The creation of process maps, as done in this study, can help identify barriers and opportunities for improvement [23, 24], and shows that there are multiple opportunities to enhance the effectiveness and efficiency of telemedicine encounters. Specific weaknesses identified in this study include challenges with scheduling, lack of electronic health record (EHR) interoperability, absence of pre-encounter planning, and problems with post-encounter information exchange.

Scheduling of telemedicine encounters in study NHs was highly de-centralized and often required multiple conversations between the providers office and NH staff before an encounter could be successfully scheduled. Scheduling is complicated if the residents HCPOA desires to be involved in the encounter. A more effective scheduling process is needed to reduce redundancies and ensure consistency [25]. For example, some NHs ended up assigning specific time slots for different primary care physicians for purposes of scheduling telemedicine visits to avoid the ongoing challenge of scheduling these encounters. This approach aligns with prior research recommending that telemedicine encounters be conducted during dedicated times of the day rather than scheduling these visits in an ad hoc fashion [26]. However, this approach focuses mainly on convenience for the NH and provider and not the residents HCPOA. Further research into the development of a comprehensive telemedicine encounter system that includes the HCPOA is needed.

Another major finding of this study was issues related to information exchange. While NHs report widespread adoption of electronic health records [27–29], interoperability remains a significant challenge. From a NH perspective, interoperability refers to the ability to electronically send and receive data from other healthcare organizations; integrate the data received into the NH EHR; and search the data from other organizations [27]. The absence of a EHR with full interoperability capability could be a barrier to sufficient information exchange between the NH and hospital or physician office EHR. Our findings confirmed the presence of interoperability barriers.

Providers with remote access to hospital and NH EHRs reported greater flexibility in gathering pertinent resident information and a perception they were more prepared for a specific telemedicine encounter. Providing clinicians with remote access to NH EHRs, particularly if the information accessed by these individuals is structured appropriately, may allow them to independently prepare for telemedicine encounters and reduce depdendence on NH staff to curate and transmit needed information about a resident’s health status. Our findings related to barriers with information exchange has been confirmed by other researchers [17, 30]. Thus, future work focused on expanding the use of telemedicine in NHs should focus on how to better leverage NH EHRs to support efficient and effective information exhange.

Even with effective electronic information exchange, there will always be a need for direct inter-professional communication. When on-site, providers are usually able to connect with the bedside nurse to obtain critical information not documented in NH health records. Accessing this information is more challenging when the provider is off-site. Conducting a pre-encounter huddle can create a structured opportunity for exchange of non-documented information and may enhance the quality of a given telemedicine encounter [26, 31]. Nevertheless, only half of the telemedicine encounters observed in this study were preceded by a huddle. Telemedicine encounters conducted without a pre-encounter huddle were typically more complex. In these cases, the provider often needed to conduct a post-encounter follow-up telephone call to obtain critical pieces of information, which involved multiple phone calls and frequently led to delays in initiating changes to the resident care plan. Future studies should focus on identifying the impact of pre-encounter huddles on telemedicine encounter outcomes, situations were they are most impactful (e.g., primary care versus sub-speciality encounters), and their optimal structure (i.e., who, when, what).

In the current study, the facilitation of telemedicine encounters was led by clinical as well as non-clinical staff. When clinical staff were not acting as the telefacilitator, or encounters were scheduled back-to-back, additional workflow steps were required. This differs from other findings which suggest that the presence of clinical NH staff is a facilitator in the telemedicine encounter delivery [17]. In our study, a majority of telemedicine encounters facilitated by clinical staff involved follow-up telephone calls to obtain additional information from their bedside nurse and initiate changes to resident care plans. Future studies of telemedicine encounters in NHs should focus on the role and training of individuals who facilitate telemedicine encounters, and identify strategies to maximize information exchange between providers and NH staff and reduces their respective workloads.

### Limitations

This study has several potential limitations. Study findings were based on interviews and observations from two NHs. While the telemedicine encounter process was similar in both study NHs, this may not be the case in other NHs that implement telemedicine differently. Future research should explore the structure of the telemedicine process across multiple NHs to establish congruence on the actual process steps. Staff availability after a direct observation, often due to staffing or other required clinical care, limited our ability to interview NH staff directly after the telemedicine encounter. As such, we were not always able to capture NH staff perceptions in real-time. Future studies should attempt to address this gap. Although resident interviews were planned, obtaining consent was problematic therefore the decision was made to eliminate NH resident interviews. As a result, it is unclear how NH residents and/or their family members viewed telemedicine as an approach to conduct a clinical encounter. Future studies of telemedicine encounters in NHs should examine their perspectives.

## Conclusion

The COVID-19 pandemic increased the use of telemedicine in NHs. Given public acceptance of telemedicine as a care delivery model, expanding the use of telemedicine beyond the COVID-19 pandemic has the potential to improve the quality of care in NHs if the telemedicine encounter is designed and used properly. The results of the current study show the telemedicine process in NHs is a complex multi-step process and identified a number of areas for improvement, including simplifying encounter scheduling and enhancing the quality of information exchange. Additional studies, such as this one, that focus on the work system from the perspective of the NH will be needed in order to enhance the efficiency and effectiveness of telemedicine encounters in NHs.

## Electronic supplementary material

Below is the link to the electronic supplementary material.

Supplementary Material 1

## Data Availability

The datasets used and/or analyzed during the current study are available from the corresponding author on reasonable request.

## Abbreviations

CMS: Center for Medicare and Medicaid Services
COVID-19: Coronavirus Disease 2019
EHR: Electronic Health Record
HCPOA: Healthcare Power of Attorney
NH: Nursing Homes
PI: Principal Investigator
SEIPS: Systems Engineering Initiative for Patient Safety
