# Supplementary material for: Characteristics of telemedicine workflows in nursing homes during the COVID-19 pandemic
Source: BMC Health Serv Res. 2023 Mar 29;23:301. doi: 10.1186/s12913-023-09249-2 (PMC10052227; doi:10.1186/s12913-023-09249-2)
Supplement: Supplementary file 1 — Supplementary Material 1 [file 12913_2023_9249_MOESM1_ESM.pdf]

Appendix A. Interview Guide: APPs & MDs

Thank you so much for participating in this interview today. We really appreciate it. This study is focused on evaluating the facilitators and barriers to the implementation of telehealth in nursing homes. We hope that you will help us identify these facilitators and barriers to improve telehealth in nursing homes across Wisconsin.

In this interview I'll ask you to walk me through a typical telehealth visit in your facility by breaking the visit into three parts: preparing for a telehealth visit, performing the visit, and the steps you take after the telehealth visit is complete. Additionally, I'll ask you about the tasks you perform, the tools you use, and the environment you work in.

This session will be recorded, but your name and any other names you use during the recording will be removed by the transcriptionist so you will have complete anonymity. Is it okay for me to start recording now?

What are the steps you go through to prepare for a telehealth visit?

Person

1. Who usually initiates the need for the visit?

- How does that get set up with you?

2. Who is usually involved when you are preparing for a telehealth visit?

- Provider
- Provider Admin Staff
- **Telemedicine Coordinator/Scheduler? Who is that for your facility?**
- SNF staff (CNA, RN, LPN, Social Worker) Other:
- Resident, Resident's family
- IT

Tasks

3. Walk me through the tasks that you perform in preparation for a telehealth visit.

- Family communication/coordination
- Chart Review
- Outside consults
- Tool acquisition (telehealth cart, iPad)
- Resident travel
- Labs/tests/Meds

- What are the tasks that others perform in preparation for the visit?
- How do these tasks compare with your pre-covid visits with residents?

Tools

4. What tools do you use to prepare for your telehealth visit?

- iPad
- Notebook
- telehealth cart
- Medical equipment
- Phone
- PPE
- Other:

Organization

5. What characteristics of the nursing homes facilitate your tasks getting done in preparation for the telehealth visits?

- Staffing
- Dedicated telehealth staff or room
- training
- technology
- other

6. What characteristics of the nursing homes inhibit your tasks getting done in preparation for the telehealth visits?

- Staffing
- Dedicated telehealth staff or room
- Training
- Technology
- Other:

7. In preparation for the telehealth visits in the facilities that you cover, what characteristics of your practice facilitate your tasks getting done?

8. In preparation for the telehealth visits in the facilities that you cover, what characteristics of your practice inhibit your tasks getting done?

Thank you for sharing. Now let's move on to ***the telehealth visit*** *itself. We would like to understand the process of a typical telehealth visit in the facilities you cover. Thinking back to a recent visit, walk me through the process starting with when the call was initiated until the call ended.*

Person

9. Who is usually involved in the actual telehealth visit?

- Provider
- Provider Admin Staff
- SNF staff (CNA, RN, LPN, Social Worker) Other:
- Telemedicine Coordinator?
- Resident, Resident's family
- IT

Tasks

10. Now can you walk me through the tasks that you perform in a telehealth visit?

- What are the tasks that others perform?
- How do these tasks compare with your pre-covid visits with residents?

Tools

11. What additional resources, supplies, or tools were needed during the telehealth visit?

- iPad
- Notebook
- Telehealth cart
- Medical equipment
- Phone
- PPE
- Other:

Organization

12. What characteristics of the nursing homes facilitate your tasks getting done during your telehealth visits?

- Staffing
- Dedicated telehealth staff or room

- Training
- Technology
- Other:

13. What characteristics of the nursing homes inhibit your tasks getting done during the telehealth visits?

- Staffing
- Dedicated telehealth staff or room
- Training
- Technology
- Other:

14. What parts of each of your facility's process are helpful in successfully accomplishing your tasks during the visit?

15. What systems or processes inhibit getting your tasks done during the telehealth visit?

Thank you for your comments. Now let's move on to ***activities post telehealth visit in the facilities you cover****. What are the steps you take right after a telehealth visit has ended?*

Person

16. Who is usually involved in a post-telehealth visit?

- Who initiates follow-up care, meds, orders, lab tests, referrals, new appointments?
- Provider
- Provider Admin Staff
- SNF staff (CNA, RN, LPN, Social Worker) Other:
- Telemedicine Coordinator?
- Resident, Resident's family

Tasks

17. What are the tasks that you perform after a telehealth visit?

- What are the tasks that others perform?
- How is information shared after the visit? (PCP, Unit Staff, Family)
- How/where is documentation completed?
- How do these tasks compare with your pre-covid visits with residents?

Tools

18. What tools do you need to use after your telehealth visit?

- iPad
- Notebook
- telehealth cart
- Medical equipment
- Phone
- PPE
- Other

Organization

19. What are the characteristics of your nursing home that support your post-telehealth tasks getting done?
20. What are the characteristics of your facility that inhibit your post-telehealth tasks getting done?
21. What parts of each of your facility's process are helpful in successfully accomplishing your tasks after the visit?
22. What systems or processes inhibit getting your tasks done after the telehealth visit?

Now we'd like you to think of the impact of providing telemedicine with special populations.

23. How does the telehealth visit differ for residents with cognitive or physical limitations?

24. How does involvement of the family or the resident power of attorney influence the telehealth visit? (prep, during, follow-up)

25. How would you change the process related to the telehealth visit in your facility to make it more beneficial to patients with special needs?

Now I'd like you to think back on how telemedicine has affected your role in your facility.

Reflection:

26. What have been the most beneficial changes with telemedicine?

- For you (workload, etc.)
- For Staff/Providers
- For residents/resident family members
- Cost (financial issues)
- Regulatory
- Staffing
- Time

27. What have been the biggest challenges with telemedicine?

- For you (workload, etc.)
- For Staff/Providers
- For residents/resident family members
- Cost (financial issues)
- Regulatory
- Staffing
- Time

28. What one thing would make the process work better for you if it could be done/changed?

That was my last question. Thank you so much for taking time of your day to speak with us. We really appreciate it. Now I have a brief demographics survey for you to fill out.

**Subj ID**	

Appendix B. Interview Guide for NH Staff

Thank you so much for participating in this interview today. We really appreciate it. This study is focused on evaluating the facilitators and barriers to the implementation of telehealth in nursing homes. We hope that you will help us identify these facilitators and barriers to improve telehealth in nursing homes across Wisconsin.

In this interview I'll ask you to walk me through a typical telehealth visit in your facility by breaking the visit into three parts: preparing for a telehealth visit, performing the visit, and the steps you take after the telehealth visit is complete. Additionally, I'll ask you about the tasks you perform, the tools you use, and the environment you work in.

This session will be recorded, but your name and any other names you use during the recording will be removed by the transcriptionist so you will have complete anonymity. Is it okay for me to start recording now?

What are the steps you go through to prepare for a telehealth visit?

<u>Person</u>

1. Who initiates the need for the telehealth visit?
   - How does that get set up?
   - Who assists with the telehealth visit (getting equipment/setting it up/being in room)?

2. Who is typically involved when you are preparing for a telehealth visit?

   - Provider
   - Provider Admin Staff
   - SNF staff (CNA, RN, LPN, Social Worker) Other:
   - Telemedicine Coordinator?
   - Resident, Resident's family
   - IT

<u>Tasks</u>

3. Walk me through the tasks that you perform in preparation for a telehealth visit. (Resident travel, family communication/coordination, tool acquisition, chart review, labs/tests, outside consults, meds, PPE)

- What are the tasks that others perform?
- How do these tasks compare with your pre-covid visits with residents?

<u>Tools</u>

4. What tools are used to prepare for the telehealth visit? (iPad, computer, notebook, telehealth cart, medical equipment, phone) Other:

<u>Organization</u>

5. In preparing for a telehealth visit, what does your facility do to help you accomplish your tasks/goals? (Leadership support, funds to purchase equipment, protocols, dedicated staff)

6. What are the barriers in your facility that inhibit preparing for telehealth visits?

Thank you for sharing. Now let's move on to ***the telehealth visit*** *itself. We would like to understand the process of a typical telehealth visit in your facility. Thinking back to a recent visit, walk me through the process starting with when the call was initiated until the call ended*.

<u>Person</u>

7. Who is usually involved in a telehealth visit?

- Provider
- Provider Admin Staff
- SNF staff (CNA, RN, LPN, Social Worker) Other:
- Telemedicine Coordinator?
- Resident, Resident's family
- IT

<u>Tasks</u>

8. Now can you walk me through the tasks that you perform in a telehealth visit?

- What are the tasks that others perform?
- How do these tasks compare with your pre-covid visits with residents?

<u>Tools</u>

9. What additional resources, supplies, or tools were needed during the telehealth visit?

(iPad, Notebook, telehealth cart, medical equipment, phone, PPE) Other:

<u>Organization</u>

10. What parts of your facility's process are helpful in successfully accomplishing your tasks during the visit?

11. What systems or processes inhibit getting your tasks done during the telehealth visit?

Thank you for your comments. Now let's move on to ***activities post telehealth visit****. What steps do you take immediately following a telehealth visit?*

<u>Person</u>

12. Who is usually involved in a post-telehealth visit?

- Who initiates follow-up care, meds, orders, lab tests, referrals, new appointments?
- Provider
- Provider Admin Staff
- SNF staff (CNA, RN, LPN, Social Worker) Other:
- Telemedicine Coordinator?
- Resident, Resident's family

<u>Tasks</u>

13. What are the tasks that you perform after a telehealth visit?

- What are the tasks that others perform?
    - How is info shared after the visit? (PCP, Unit Staff, Family)
    - How/where is documentation completed?
    - How do these tasks compare with your pre-covid visits with residents?

Tools

14. What tools do you need to use after your telehealth visit?

(iPad, Notebook, telehealth cart, medical equipment, phone, EHR/chart) Other:)

Organization

15. What are the characteristics of your facility that support your post-telehealth tasks getting done?

16. What are the characteristics of your facility that inhibit your post-telehealth tasks getting done?

Now we'd like you to think of the impact of providing telemedicine with special populations.

17.How does the telehealth visit differ for residents with cognitive or physical limitations?

18. How does involvement of the family or the resident power of attorney influence the telehealth visit? (prep, during, follow-up)

19. How would you change the process related to the telehealth visit in your facility to make it more beneficial to patients with special needs?

For SW/Nurse Case Manager ONLY

Now I have a few questions about how telehealth training was implemented in your facility.

TELEHEALTH TRAINING

- Who did your telehealth training?
- When were you trained on telehealth and how much time did it take?
- Did you feel the training you received was adequate?
  - Did you feel that you were adequately prepared?
- How do you feel now?
  - Would you benefit from additional training at this point?

TELEHEALTH Visits Per Month

- Could you tell us how many telehealth visits you perform per month?
- How many of those require you to set up and leave? How many of those require you to remain for the entire visit?

Now I'd like you to think back on how telemedicine has affected your role in your facility.

Reflection:

20. What have been the most beneficial changes with telemedicine?

- For you (workload, etc.)
- For Staff/Providers
- For residents/resident family members
- Cost
- Regulatory
- Staffing
- Time

21. What have been the biggest challenges with telemedicine?

- For you (workload, etc.)
- Platform and Connectivity Stability
- For Staff/Providers
- For residents/resident family members
- Cost (financial issues)
- Regulatory
- Staffing
- Time

22. What, in hindsight, do you think would’ve make the telehealth implementation process work better for you if it could be done/changed?

That was my last question. Thank you so much for taking time out of your day to speak with us. We really appreciate it. Now I have a brief demographic survey for you to fill out.

Appendix C. Interview Guide for NH Administrator, Director of Nursing and Medical Director

Thank you for participating in this interview today. We really appreciate it. This study is focused on how telehealth is being used and implemented in your facility with the goal of trying to make it work better. The knowledge gained from this study will help us improve telehealth in your facility and those across Wisconsin.

For this interview we're going to ask you questions about how your telehealth program is being used and implemented in your facility and some of the issues you have encountered. This session will be recorded, but any names that you use will be redacted by the transcriptionist to protect your anonymity. It is okay for me to press record? Great, let's get started. My first question is:

1. In thinking about resident visits pre-COVID and post-COVID resident care, how has resident care changed?
    - In-person
    - Telehealth
    - Wound care visits
    - PT, OT, Behavior, hospice
    - Specialist care
2. To what extent was your facility using telehealth in your facility pre covid?
    - What types of visits is telehealth being used for in your facility?
3. Can you describe what is involved with performing a telehealth visit in your facility? How does this differ based on the type of visit?

Person: Who is involved in the provision of telemedicine (pre-process, during, post)?
Tasks: What are the tasks that need to be performed (pre/during/post)?
Tools: What tools are needed (pre/during/post)?
Org: What are the characteristics of your facility that support your post-telehealth tasks getting done?
Org: What are the characteristics of your facility that inhibit your post-telehealth tasks getting done?

4. What was involved with implementing telehealth visits in your facility?
    - Training of staff on telehealth (When did it occur? Who did the training?)
    - Adequacy of training
    - Need for further training?
5. What have been the benefits of telehealth visits to your facility? To your residents?
    - For you (workload, etc.)
    - For Staff/Providers
    - For residents/resident family members
    - Less travel- Other Services
    - Cost
    - Regulations
    - Equipment/training
    - Time
6. What have been the challenges encountered with implementing and using telehealth encounters in your facility?

- For you (workload, etc.)
- For Staff/Provider
- For residents /resident family members
- Regulations
- Cost
- Staffing
- Equipment/training
- Time

Now I'd like you to think back on how COVID-19 has affected your role in your facility.

Reflection:

7. How has your role changed in your facility, pre-COVID to post-COVID in terms of telehealth?
   - How has the wider use of telehealth in your facility changed your role as a Medical
8. Are you anxious to get back to in-person visits and limit telehealth once COVID crisis is over? Why? Why not?

We have two quick questions for you about staff coverage in your facility that we wanted to clarify.

9. How many provider groups are in your facility?
   - What are they?
   - What percent of your residents are followed by UW APP's?
   - How are the remainder followed?
10. Is telehealth utilized for any after-hours or emergency care? If yes, describe how it's being used in your facility.

*That was my last question. [**If DON, read below**]*

FOR DON:
Before we go, I wanted to ask if it's okay to contact the RCNs and the SW to talk about the process they use to schedule and coordinate telehealth visit and would she let them know to expect an email from us.

I also have a couple of clarifications about telehealth visit observations and we wanted to get your feedback.

We are going to observe a few visits at your facility and would like to recruit staff and patients to observe and then interview afterwards. These will not be recorded, and our research staff will NOT speak or participate in the visit itself. The visits will be selected by APPs. We would like to identify staff involved in these for brief interviews. We'd like to arrange a brief after-visit to clarify anything we don't understand from the visit and elicit their feedback on the telehealth process.

How can we find out when the visits are happening, who is involved in facilitating the telehealth visit? How can we make sure the patient is warned in advance? How can we contact patients and/or staff prior/post?

Thank you so much for taking time out of your day to speak with us. We really appreciate it. Now we have a quick demographic survey for you if you have a few minutes.

SUBJ ID	

Appendix D. Telemedicine Encounter Checklist

Subj ID:
# Observers:
Coder initials:

PARTICIPANTS

1. **Provider Type:** ☐ MD ☐ APP
2. **Who was the telefacilitator?** ☐RCM ☐ MED Rec Staff ☐ SW ☐ Other (list):

PRE-HUDDLE

3. **Did a Pre-huddle occur?** ☐Yes ☐No (**If NO, SKIP questions below)**
4. **Who else was involved (besides the provider and the telefacilitator)**
   ☐No one ☐RN/LPN ☐SW ☐ Other (list):
5. **What was discussed?**
   ☐ Conversation about active medical problem (legs more swollen, high BP, weight gain)
   ☐ Vitals (Only include temperature, blood pressure, pulse, respiration, oxygen saturation)
   ☐ Meds
   ☐ Labs (including blood sugar)
   ☐ Care coordination/planning issues (had PT, appointments)
   ☐ Psycho/social (depression, lack of family support)
   ☐ Other (list):
6. **Did all of the provider's needs appear to get addressed** ☐Yes ☐No ☐ Not sure
7. **Were patient orders conveyed to nursing home staff (medications, lab orders, etc.) during the pre-huddle?**
   ☐No ☐Yes, (if yes, describe):
8. **Pre-huddle: Describe other notable things (tasks/tools/org unique to telehealth):**

VISIT

9. **Type of Visit:** ☐ Admission ☐Acute ☐Compliance ☐Discharge ☐ Not Sure
10. **Persons involved in visit**: ☐ Provider ☐RCM ☐ Med Rec Staff ☐RN/LPN ☐Resident ☐ Resident's family member/HPOA ☐ Other (list):
11. **PPE used by telefacilitator?** ☐Yes ☐No ☐ Not Sure
12. **PPE used by patient?** ☐Yes ☐No ☐ Not Sure
13. **Describe positioning of Provider on video:**
14. **Describe positioning of Resident on video (including any changes in positioning during the visit):**
15. **Telefacilitator used as resource to provider?** ☐Yes ☐No
16. **What tasks did the telefacilitator perform during the encounter? (Check all that apply)**
    ☐ Provided assistance for physical exam (show legs, assist with standing)
    ☐ Performed aspects of the clinical exam. **If YES: What exams?**
    ☐ Provided history that otherwise wasn't provided by the resident
    ☐ Facilitated communication from resident to provider
    ☐ Facilitated communication from provider to resident.
    ☐ Other:

17. **Role of Resident family member/HPOA in encounter**
    ☐ Not Applicable (not present for encounter)
    ☐ Knowledge taking (e.g., spouse wants an update on what is going on with the resident)
    ☐ Knowledge giving (e.g., spouse of resident is giving the provider information they need to complete their history)
    ☐ Decision making (e.g., spouse of resident is making preferences on behalf of the resident known)
18. **What was the participants' level of engagement in the visit**?
    ☐ Asking and responding to questions
    ☐ Only responding to questions
    ☐ Unable or unwilling to respond to questions
19. **Patient has hearing difficulty?** ☐Yes ☐No ☐ Not Sure
20. **Patient is confused?** ☐Yes ☐No ☐ Not Sure
21. **Patient has neurologic deficit interfering with speech?** ☐Yes ☐No ☐ Not Sure
22. **Were patient orders conveyed to nursing home staff (medications, lab orders, etc.) during the visit?**
    ☐No ☐Yes, (if yes, describe):
23. **Visit: Describe other notable things (tasks/tools/org unique to telehealth):**

POST-HUDDLE

24. **Did a post-huddle occur?** ☐Yes ☐No

If YES:

25. **Was additional information gathered after encounter?** ☐ Yes ☐No
26. **What tasks were performed in the post-huddle?**
    ☐Additional history taking performed
    ☐Resident care orders provided
    ☐Other resident care coordination tasks discussed or assigned. If YES please specify: ___________
27. **Who was involved in performing the above tasks?**
    ☐ Telefacilitator ☐ Someone else (list): __________
28. **Did all of the provider's needs appear to get addressed?** ☐ Yes ☐ No ☐ Not sure
29. **Post-Huddle: Describe other notable things (tasks/tools/org unique to telehealth):**

DELAYED-FOLLOW-UP**

30. **Did the provider indicate that they were going to call back* (follow up with the facility) during the visit?** ☐Yes ☐No
31. **If YES, what activities did they plan to perform?**
    ☐ Gather additional information (from NH staff)
    ☐ Submit orders for resident
    ☐ Discuss other care coordination (FU with NH staff, other providers, or call resident family member)
    ☐ Unclear purpose

GENERAL

32. **Quality of Audio***

☐Excellent (5) ☐ Good (4) ☐Fair (3) ☐ Poor (2) ☐Very Poor (1)

33. **Quality of Video***
    ☐Excellent (5) ☐Good (4) ☐Fair (3) ☐ Poor (2) ☐ Very Poor (1)
34. **Was re-positioning of the video device or the patient necessary due to patient positioning during the encounter** (provider asked patient or telefacilitator to move the device or patient, or the telefacilitator did this on their own)**?** ☐Yes ☐No ☐ Not Sure
35. **Did any connection or other technology issues occur during the telehealth visit?**
    ☐Yes ☐ No ☐ Not Sure
36. **If YES, describe:**
37. **How long was the telehealth visit?** ________ (min) [**Use range of minutes- only those actually listed]**
38. **Did the provider conduct more than one telehealth visit in addition to this visit?** ☐ Yes ☐No
39. **If YES, how many:**
40. **General: Describe other notable things (tasks/tools/org unique to telehealth):**

Glossary

MED Rec Staff- Medical Record-keeping Staff

****Delayed Follow-up**: For Following up (call back): This is not for submitting notes, this is to follow up for information or orders with the nursing home.

AUDIO & VIDEO QUALITY SCALES (Absolute category rating) *

Audio

Excellent (5): Perfect audio quality without any glitches or issues in hearing from either party.
Good (4): 1-2 interruptions (static/glitches) that caused minor interruptions in care and communication.
Fair (3): Several glitches/interruptions that caused moderate interruptions in care and communication.
Poor (2): Many interruptions that caused severe lags in communication and missed opportunities for delivery of care to the resident
Very Poor (1): Call dropped- unable to connect, unable to communicate or conduct most of the visit with the resident.

Video

Excellent (5): Perfect visual quality without any glitches or delays from either party.
Good (4): 1-2 interruptions (lag or frozen screen) that caused minor interruptions in care and communication.
Fair (3): Several interruptions or more prolonged interruptions (lag, frozen screen) that caused moderate interruptions in care and communication.
Poor (2): Many interruptions that caused severe lags in communication and missed opportunities for delivery of care to the resident.
Very Poor (1): Video is not working and unable to connect visually with resident.
